# Metal Preferences of Zinc-Binding Motif on Metalloproteases

**DOI:** 10.4061/2011/574816

**Published:** 2011-05-11

**Authors:** Kayoko M. Fukasawa, Toshiyuki Hata, Yukio Ono, Junzo Hirose

**Affiliations:** ^1^Department of Hard Tissue Research, Graduate School of Oral Medicine, Matsumoto Dental University, Shiojiri, Nagano 399-0781, Japan; ^2^Faculty of Pharmacy and Pharmaceutical Science, Fukuyama University, Gakuen-cho, Fukuyama 729-0292, Japan

## Abstract

Almost all naturally occurring metalloproteases are monozinc enzymes. The zinc in any number of zinc metalloproteases has been substituted by some other divalent cation. Almost all Co(II)- or Mn(II)-substituted enzymes maintain the catalytic activity of their zinc counterparts. However, in the case of Cu(II) substitution of zinc proteases, a great number of enzymes are not active, for example, thermolysin, carboxypeptidase A, endopeptidase from *Lactococcus lactis*, or aminopeptidase B, while some do have catalytic activity, for example, astacin (37%) and DPP III (100%). Based on structural studies of various metal-substituted enzymes, for example, thermolysin, astacin, aminopeptidase B, dipeptidyl peptidase (DPP) III, and del-DPP III, the metal coordination geometries of both active and inactive Cu(II)-substituted enzymes are shown to be the same as those of the wild-type Zn(II) enzymes. Therefore, the enzyme activity of a copper-ion-substituted zinc metalloprotease may depend on the flexibility of catalytic domain.

## 1. Introduction

Proteolytic enzymes are recognized by their catalytic type, that is, aspartic, cysteine, metallo, serine, threonine, and others as yet unclassified. The largest number of proteolytic enzymes are classified as metalloproteases [1]. Almost all metalloproteases contain one or two zinc ions, and several enzymes contain one or two cobalt or manganese ions. The HExxH motif forming an *α*-helix is well conserved in many monozinc enzymes as the active site in which the two histidine residues coordinate with the zinc ion [2]. Some other monozinc proteases have different zinc-binding motifs, for example, HxxE(D)-aa_n_-H in the carboxypeptidase family or HxD-aa_12_-H-aa_12_-H in the matrix metalloprotease family [2]. Dipeptidyl peptidase (DPP) III also has a unique zinc-binding motif, which was classified as family M49 in 1999 by MEROPS (peptidase database) after rat DPP III had been cloned and the HELLGH motif of DPP III was identified as an active site coordinated with a zinc ion [3, 4]. Although the motif HELLGH could not be found in any other metalloproteases, it exists in three kinds of monooxygenases (tyrosine, phenylalanine, and tryptophan hydroxylases) as an iron-binding site, as revealed by a search of the NBRF-PIR protein sequence database. 

Zinc atoms in several zinc metalloproteases, for example, astacin [5], carboxypeptidase A [6], and thermolysin [7, 8], have been substituted by other divalent cations to probe the role of the metal for catalysis and structure. Some of these enzymes, for example, DPP III and astacin, were shown to have high metal substitution tolerance by metal substitution studies [9]. However, it is difficult to determine the relationship between the metal tolerance and the metal coordination structure of zinc metalloproteases.

Here, we show the metal coordination structure of the unique zinc-binding motif of DPP III, in which the zinc-binding motif is stabilized by several hydrogen bonds with acidic amino acid residues surrounding the zinc-binding motif, in order to clarify the relationship between the metal tolerance and the structure of the zinc-binding domain. The metal tolerances of both DPP III and del-DPP III, whose active site converts into a normal zinc-binding motif (HExxH), are shown here and compared with those reported for other metalloproteases [10]. Finally, we discuss the relationship between the catalytic activities and metal coordination structures of metal-substituted enzymes.

## 2. Identification of a Zinc-Binding Motif in DPP III

We start with the identification of the zinc-binding motif in DPP III, which will be used for further investigation of the relationship between the metal tolerance and the metal coordination structures of DPP III. The deduced amino acid sequences from cDNA for human, rat, and fruit fly DPP IIIs are 723–738 amino acids long and conserve the amino acid sequence HELLGH-aa_52_-E [3, 11, 12], which resembles the HExxH-aa_n_-E zinc-binding motif conserved in many metalloproteases, such as thermolysin [13] and leukotriene A_4_ hydrolase [14]. Site-directed mutagenesis was performed on rat DPP III in order to testify that the HELLGH-aa_52_-E is a zinc-binding domain. Site-directed mutagenesis studies have clearly shown that the H450Y, H455Y, and E508A mutants, which lack zinc ions, lose their catalytic activity [4]. Replacement of Glu^451^ in these mutants with an alanine or an aspartic acid restores a mol of zinc ion per mol of protein but does not restore catalytic activity [4]. These results show that the H^450^ELLGH-aa_52_-E^508^ motif is a catalytic domain of which His^450^, His^455^, and Glu^508^ are ligands of a zinc ion and of which Glu^451^ is a catalytic amino acid residue, in the same way that the H^142^ExxH-aa_19_-E^166^ motif of thermolysin is a catalytic domain of which His^142^, His^146^, and Glu^166^ are ligands of a zinc ion and Glu^143^ is a catalytic amino acid residue. 

The 1.95-Å crystal structure of yeast DPP III representing a prototype for the M49 family of metalloproteases was resolved by Baral et al. [15]. It shows a novel protein fold with two domains forming a wide cleft containing the catalytic metal ion. However, the three-dimensional structure of zinc coordination (His^460^, His^465^, and Glu^517^) and the catalytic active (Glu^461^) residues are structurally conserved, similar to those presented in many metalloproteases, such as thermolysin [13]. The HELLGH motif and the third ligand (Glu^517^) of DPP III construct a helix *α*14 and a helix *α*16, respectively [15]. The 3D structure of DPP III is similar to that of thermolysin [13] or leukotriene A_4_ hydrolase [16], the zinc-binding domain of which is constructed of two *α*-helixes, for HExxH (containing two zinc ligands) and xNEx (third ligand). 

**Figure 1:**
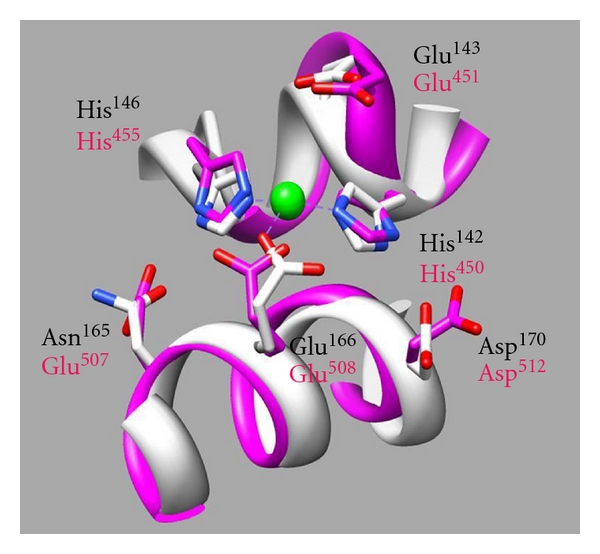
The superimposition of the active sites of rat DPP III and thermolysin. Zinc ion is shown as a green sphere, and amino acid side chains are shown as sticks colored red for oxygen and blue for nitrogen. Metal coordinates in light blue and hydrogen bonds in yellow are indicated by dashed lines. Carbon atom and amino acid chain are shown colored white for thermolysin and magenta for DPP III. Metal coordination bonds are indicated by light blue dashed lines.

Figure 1 shows the superimposition of the active sites of rat DPP III and thermolysin. The helix *α*14 of DPP III has a slightly larger loop than that of thermolysin, and the glutamic acid on the motif comes close to zinc ion comparing with the glutamic acid on the normal helix of thermolysin [13, 17].

## 3. Stabilization of the Coordination between Ligands and Metal

**Figure 2:**
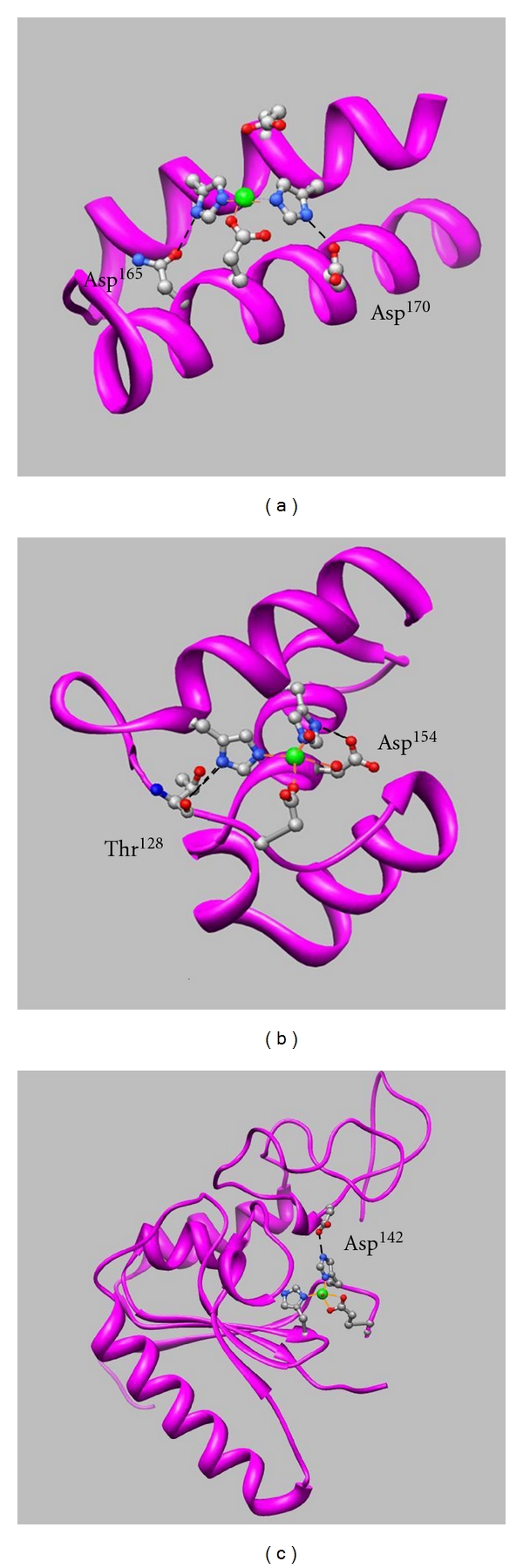
Three-dimensional structures of the catalytic domain models for thermolysin ((a): PDB 1KEI), peptidyl-Lys metallopeptidase ((b): PDB 1GE6), and carboxypeptidase A ((c): PDB 1YME). The zinc ion is shown as a green sphere, and amino acid side chains are shown as sticks colored red for oxygen, blue for nitrogen, and silver for carbon. Hydrogen bonds are indicated by dashed lines.

**Figure 3:**
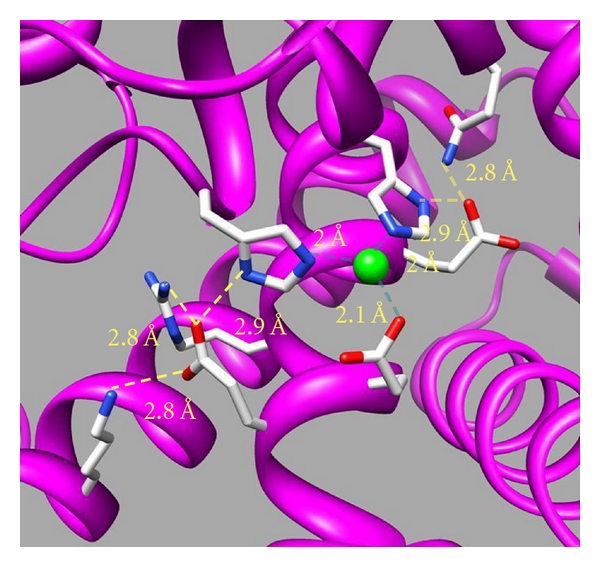
Molecular modeling of the catalytic site of rat DPP III. The model was generated as a template of the human DPP III crystal structure [39]. The zinc ion is shown as a green sphere, and amino acid side chains are shown as sticks colored red for oxygen, blue for nitrogen, and white for carbon. Metal coordinates in light blue and hydrogen bonds in yellow are indicated by dashed lines.

In the 3D structural model of the zinc-binding domain of many zinc enzymes—neprilysin [18], thermolysin [13], carboxypeptidase A [19], leukotriene A_4_ hydrolase [16], aspzincin [20], and DPP III [17]—the His, His, and Glu residues that coordinate with the zinc ion are engaged in hydrogen bonds with one or two acidic amino acid residues (Glu or Asp) or other carbonyl oxygen atoms (Table 1). 3D structural models of catalytic domains of thermolysin (PDB: 1KEI), peptidyl-Lys metallopeptidase (PDB: 1GE6), and carboxypeptidase A (PDB: 1YME) are shown in Figure 2. In thermolysin (a), the oxygen atoms of Asp^165^ and Asp^170^ are engaged in hydrogen bonding with the nitrogen atoms of His^146^ and His^142^, respectively. Asp^154^ and Thr^128^ of peptidyl-Lys metalloendopeptidase (b) and Asp^142^ of carboxypeptidase A (c) are also engaged in hydrogen bonding with His^117^, His^121^, and His^69^, respectively. It was proved through the mutational studies of rat DPP III that this network of hydrogen bonds close to the zinc-binding motif plays an important role in stabilizing the coordination of the zinc ion to the protein [17]. The hydrogen bonds surrounding the zinc-binding motif of rat DPP III are shown in Figure 3, and the kinetic parameters, zinc contents and zinc dissociation constants of the several mutants are shown in Table 2. The replacement of Glu^507^ and Glu^512^, the oxygen atoms of which bind with the nitrogen atoms of His^455^ and His^450^, respectively, increases the dissociation constants by factors of 10~10^5^ and correlatively reduces the zinc contents and enzyme activities. The hydrogen bonds between acidic amino acid residues and zinc ligands (His, His, and Glu) may stabilize the coordination of the zinc ion with the protein of the metalloprotease.

## 4. Metal Substitutions of Monozinc Metalloproteases

 Almost all metalloproteases are monozinc enzymes. Some enzymes contain two zinc ions for catalytic domains, for example, human renal dipeptidase [36], and a few are dicobalt or dimanganese enzymes, for example, *Pyrococcus furiosus* methionine aminopeptidase [37] or *Escherichia coli* proline aminopeptidase [38], respectively. 

The zinc in numerous zinc metalloproteases has been substituted by several divalent cations. The cobalt(II)- or manganese(II)-substituted enzymes showed nearly restored catalytic activity or even excess activity from apoenzyme, as seen in Table 3. 

Gomis-Rüth et al. [5] demonstrated in the metal substitution studies of astacin that Cu(II)-astacin displays enzyme activity of about 37%, while Ni(II)- and Hg(II)-astacin were almost inactive. In the crystal structure of Cu(II)-astacin, the metal ion is pentacoordinated with His^92^, His^96^, His^102^, Tyr^149^, and H_2_O, as in native Zn(II)-astacin or Co(II)-astacin; however, in the Ni(II)-astacin or Hg(II)-astacin, the metal ion is hexacoordinated with an additional solvent molecule or tetracoordinated with no ordered solvent molecule, respectively [5]. The restoration of catalytic activity in these substituted astacins was shown to be dependent on the metal coordination structure [5].

Meanwhile, almost all Cu(II)-substituted enzymes, such as thermolysin [7, 8], carboxypeptidase A [6], aminopeptidase B [22], or endopeptidase from *Lactococcus lactis* [30], show only partial activation or very low activities. The reason why these Cu(II) enzymes do not demonstrate catalytic activities may be that the coordination geometry of Cu(II) is more rigid than that of Zn(II) or Co(II). 

In the case of DPP III, Co^2+^-, Ni^2+^- and Cu^2+^-DPP IIIs showed comparable catalytic activities to Zn^2+^-DPP III; the kinetic parameters are shown in Table 4 [9]. DPP III shows high flexibility of the metal ion for the catalytic activity compared with thermolysin or aminopeptidase B. Thermolysin or aminopeptidase B is a subclan MA (E) metalloprotease containing an HExxH-aa_n_-E motif, and the 3D structure of the active domain is very similar to that of DPP III described above. The zinc ion in a subclan MA (E) metalloprotease or DPP III is tetracoordinated with three ligands (His, His, and Glu) and a water molecule. The metal-substituted (Co^2+^, Cu^2+^, or Ni^2+^) DPP III may have the same tetrahedral coordination structure as Zn^2+^-DPP III, so these enzymes are able to maintain the catalytic activity. The zinc in del-DPP III, whose active site converted into HExxH, was substituted with Co^2+^, Ni^2+^, or Cu^2+^to investigate the grounds for activation of the Cu^2+^-DPP III [10]. The Co^2+^-del-DPP III and Ni^2+^-del-DPP III showed comparable catalytic activity to that of Zn^2+^-del-DPP III, while the Cu^2+^-del-DPP III showed no catalytic activity, as in the case of thermolysin or aminopeptidase B [8–10].

 The EPR (electron paramagnetic resonance) parameters of various Cu^2+^-substituted metalloproteases are shown in Table 5. Each parameter is exactly alike between DPP III and thermolysin, aminopeptidase B, or del-DPP III [8–10, 22]. The results show that the Cu(II) coordination structures of the HExxH-aa_n_-E and HExxxH-aa_52_-E motifs are very similar. 

In the superimposition of the 3D structure models of active sites of DPP III and del-DPP III, the *α*-helix of DPP III, which is abnormally composed of 5 amino acid residues per one turn of the *α*-helix, is a larger loop than that of del-DPP III, the same as the case for the superimposition of the active sites of DPP III and thermolysin (Figure 1). The coordination geometries of the two enzymes are similar, while the position of Glu^451^, which is essential for the enzyme activity, is slightly closer to the copper ion in DPP III than in del-DPP III. The distances of oxygen atoms of the Glu^451^ residues of del-DPP III and wild-type DPP III are 4.8 Å and 3.2 Å from the zinc ion, respectively. Zn(II) coordination geometry is flexible, so both wild-type and del-DPP IIIs could have catalytic activity. However, the oxygen atom of Glu^451^ in Cu(II)-del-DPP III is not able to bind to the oxygen atom of the water molecule that is coordinated with the copper ion because the Cu(II) coordination geometry is very rigid. Therefore, the catalytic activity of Cu(II)-del-DPP III was diminished. 

Some other Cu(II)-substituted enzymes, for example, aminopeptidase Ey [21], vibriolysin [40], hyicolysin [32], and *Legionella* metalloendopeptidase [33], were shown to have enzyme activities. These enzymes are all classified in subclan MA (E), the same as thermolysin or aminopeptidase B. The metal coordination structures of these enzymes have not been shown in detail; however, the catalytic domain may be more flexible than that of thermolysin or aminopeptidase B, in the same way as Cu(II)-substituted DPP III.

## 5. Conclusions

In this paper, we compared metal flexibility with the geometry of metal coordination of metalloproteases, to investigate why DPP III shows metal tolerance. Metal substitution of Zn(II) by Co(II) or Mn(II) on metalloproteases generally maintains catalytic activity, because the metal coordination geometries of Zn(II), Co(II), and Mn(II) are flexible. Most Cu(II)-substituted enzymes could not restore the catalytic activities, because the Cu(II) coordination geometry is very rigid. However, Cu(II)-substituted DPP III showed the same catalytic activity as that of Zn(II)-DPP III. We then studied the metal flexibilities and metal coordination geometries of many metallopeptidases, especially DPP III and del-DPP III, but we could not prove a relation between the metal flexibility and the metal coordination geometry. The metal tolerance of DPP III might depend on the flexibility of the metal-binding motif, not on the metal coordination geometry. By comparison of the 3D structure of active sites of DPP III and del-DPP III, both coordination geometries are seen to be similar, while the positions of catalytic amino acid residues (Glu) on those zinc-binding motifs are slightly different. We conclude that the catalytic site of Cu(II)-DPP III could be flexible enough to form the catalytic complex, with substrate and H_2_O.

## Figures and Tables

**Table 1:** The zinc coordination residues and the residues that fix the coordination with hydrogen bonds.

Zinc metalloprotease	Coordination residues	Residues that form the hydrogen bond with the coordination residues	PDB no.
(1) Thermolysin type	(HExxH- aa_n_-E)	*α*-helix-aa_n_-*α*-helix	
Thermolysin	His^142^, His^146^ Glu^166^	Asp^170^-2.8 Å-His^142^ Asn^165^-2.8 Å-His^146^	1KEI
Vibriolysin	His^345^, His^349^ Glu^369^	Asp^373^-2.8 Å-His^345^ Asn^368^-2.8 Å-His^349^	3NQX
Staphylococcus aureus metalloproteinase	His^144^, His^148^ Glu^168^	Asn^167^-2.8 Å-His^148^ Asp^172^-2.8 Å-His^144^	1BQB
Zinc aminopeptidase	His^265^, His^269^ Glu^288^	Phe^272^(C=O)-2.9 Å-His^269^	1Z1W
Leukotriene A4 hydrolase	His^295^, His^299^ Glu^318^	Glu^325^-2.8 Å-His^295^ Gly^303^(C=O)-2.6 Å-His^299^	1SQM
Human thimet oligopeptidase	His^473^, His^477^ Glu^502^	Glu^509^-2.6 Å-His^473^	1SQM
Human neutral endopeptidase (Neprilysin)	His^583^, His^587^ Glu^646^	Asp^650^-2.9 Å-His^583^ Asp^590^-2.7 Å-His^587^	1DMT

(2) Endopeptidase type		(HExxH-aa_n_-E or D) *α*-helix-aa_n_-random coil	
Peptidyl-Lys metalloendopeptidase	His^117^, His^121^ Asp^130^	Asp^154^-2.7 Å-His^117^ Thr^128^(C=O)-2.8 Å-His^121^	1GE6

(3) Carboxypeptidase A type		*β*-sheet-aa_n_-random coil	
Carboxypeptidase A	His^69^, His^196^ Glu^72^	Asp^142^-2.7 Å-His^69^	1YME
Putative lysostaphin peptidase	His^232^, His^311^ Asp^236^	Glu^315^-2.6 Å-His^311^ Gly^216^(C=O)-2.8 Å-His^232^	2GU1

**Table 2:** Kinetic parameters for the hydrolysis of Arg-Arg-NA, zinc contents, and zinc dissociation constants of wild-type and mutated rat DPP IIIs.

Enzymes	*k*_cat_/*K*_*M*_ × 10^−4^ (M^−1^ s^−1^)	Zinc content (mol/mol of protein^a^)	Zinc dissociation constant (M) (*K*_*d*_)
Wild-type	73.6 ± 6.9	1.02 ± 0.15	(4.5 ± 0.1) × 10^−13^
E507D	22.8 ± 1.9	0.65 ± 0.07	(1.0 ± 0.2) × 10^−11^
E507A	4.43 ± 0.41	0.29 ± 0.04	(1.0 ± 0.2) × 10^−8^
E512D	21.0 ± 0.19	0.45 ± 0.06	(1.4 ± 0.1) × 10^−12^
E512A	2.45 ± 0.28	0.08 ± 0.01	(2.6 ± 0.7) × 10^−9^

^a^Values are means ± SD of two separately prepared enzymes with duplicate determinations.

**Table 3:** Reactivated Co(II) and Mn(II) enzymes substituted from apo-metalloproteases.

Clan	Subclan	Name of enzyme	Replaced Ion	Reference
MA	E	Aminopeptidase Ey	Co^2+^, Mn^2+^	Tanaka and Ichishima [21]
MA	E	Aminopeptidase B	Co^2+^	Hirose et al. [22]
MA	E	Saccharolysin	Co^2+^, Mn^2+^	Achstetter et al. [23] and Büchler et al. [24]
MA	E	Lysyl aminopeptidase	Co^2+^, Mn^2+^	Klein et al. [25]
MA	E	Oligopeptidase F	Co^2+^, Mn^2+^	Yan et al. [26] and Monnet et al. [27]
MA	E	Mycolysin (Thermolysin)	Co^2+^, Mn^2+^ Co^2+^ (200%), Mn^2+^ (10%)	Chang and Lee [28], and Holmquist and Vallee [29]
MA	E	Oligopeptidase O	Co^2+^ Mn^2+^ (50%)	Tan et al. [30] and Baankreis et al. [31]
MA	E	Hyicolysin	Co^2+^	Ayora and Götz [32]
ME	E	Legionella metalloendopeptidase	Mn^2+^ (69%)	Dreyfus and Iglewski [33]
MA	A	Epralysin	Co^2+^ (58%)	Diermayr et al. [34]
MA	M	Astacin	Co^2+^ (140%)	Gomis-Rüth et al. [5]
MA	M	MEP^a^ (*Gf* ^b^ MEP)	Co^2+^ Mn^2+^ (200%)	Nonaka et al. [35]
MA	M	*Po* ^c^ MEP	Co^2+^ (80%) Mn^2+^ (30%)	Nonaka et al. [35]

^a^Peptidyl-Lys metallopeptidase; ^b^ *Grifola frondosa*; ^c^ *Pleurotus ostreatus.*

**Table 4:** Kinetic parameters for the hydrolysis of Arg-Arg-NA and metal contents of various metal-DPP IIIs.

Enzyme	*K*_*M*_ (×10^−5^ M)	*k*_cat_ (s^−1^)	*k*_cat_/*K*_*M*_(×10^4^ M^−1^ s^−1^)	Metal content (mol/mol of protein)
Zn^2+^-DPP III	8.1 (±1.0)	7.1 (±0.2)	8.8	0.8 (±0.1)
Co^2+^-DPP III	8.2 (±0.9)	7.0 (±0.1)	8.5	1.0 (±0.1)
Cu^2+^-DPP III	9.9 (±1.1)	10.1 (±0.3)	10.2	1.1 (±0.1)

**Table 5:** EPR parameters of Cu^2+^ proteases.

	*g*_⊥_	*g_ll_*	*A_ll_* (×10^−4^ cm^−1^)
Cu^2+^-DPP III^a^	2.06	2.27	167
Cu^2+^-del-DPP III^b^	2.06	2.27	161
Cu^2+^-thermolysin^c^	2.06	2.26	163
Cu^2+^-aminopeptidase B^d^	2.06	2.27	157
Cu^2+^-carboxypeptidase A^e^	2.05	2.33	115

References ^a^[9], ^b^[10], ^c^[8], ^d^[22], and ^e^[6].
